# The genome sequence of the rosy rustic, *Hydraecia micacea *(Esper, 1789)

**DOI:** 10.12688/wellcomeopenres.17832.1

**Published:** 2022-04-07

**Authors:** Douglas Boyes, University of Oxford and Wytham Woods Genome Acquisition Lab, Darwin Tree of Life Barcoding collective, Wellcome Sanger Institute Tree of Life programme, Wellcome Sanger Institute Scientific Operations: DNA Pipelines collective, Tree of Life Core Informatics collective, Rosy Turner, Darwin Tree of Life Consortium

**Affiliations:** 1 UK Centre for Ecology and Hydrology, Wallingford, Oxfordshire, UK; 2 Independent Researcher, Cardiff, UK

**Keywords:** Hydraecia micacea, rosy rustic, genome sequence, chromosomal, Lepidoptera

## Abstract

We present a genome assembly from an individual female *Hydraecia micacea* (the rosy rustic; Arthropoda; Insecta; Lepidoptera; Noctuidae). The genome sequence is 562 megabases in span. The majority of the assembly (99.98%) is scaffolded into 32 chromosomal pseudomolecules, with the W and Z sex chromosomes assembled. The mitochondrial genome was also assembled, and is 16.3 kilobases in length.

## Species taxonomy

Eukaryota; Metazoa; Ecdysozoa; Arthropoda; Hexapoda; Insecta; Pterygota; Neoptera; Endopterygota; Lepidoptera; Glossata; Ditrysia; Noctuoidea; Noctuidae; Noctuinae; Apameini; Hydraecia; *Hydraecia micacea* (Esper, 1789) (NCBI:txid214171).

## Background

The Rosy Rustic Moth (Hydraecia micacea) is found across the northern hemisphere and was introduced into North America in the early 1900s ( Šedivý *et al*., 2010). The species is classed as vulnerable in the UK using International Union for Conservation of Nature (IUCN) criteria ( Conrad *et al*., 2006). Hydraecia micacea is mostly found in wetlands and has one generation per year with its flying period occurring approximately from mid-July until October ( Weihrauch, 2020). The species feeds on approximately 50 different plant species and is a damaging pest to weeds and several crop species including potatoes, cereals and hops due to the moth’s stem boring larvae ( Gryndler *et al*., 2008; Šedivý *et al*., 2010). The genome of this species will give an insight into H. micacea’s herbivory habits allowing for more targeted pest control methods ( You *et al*., 2013).

## Genome sequence report

**Figure 1.**
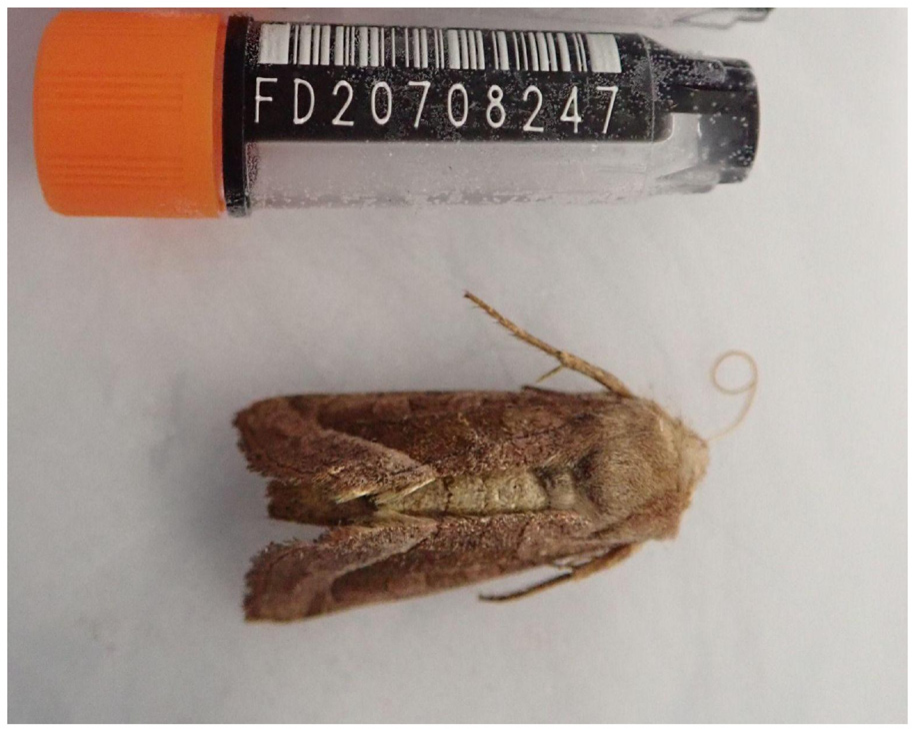
Image of the *Hydraecia micacea* (ilHydMica1) specimen taken during preservation and processing.

The genome was sequenced from one female *H. micacea* ( Figure 1) collected from Wytham Woods, Oxfordshire (Biological vice-county: Berkshire), UK (latitude 51.772, longitude -1.338). A total of 30-fold coverage in Pacific Biosciences single-molecule long reads and 79-fold coverage in 10X Genomics read clouds were generated. Primary assembly contigs were scaffolded with chromosome conformation Hi-C data. Manual assembly curation corrected 11 missing/misjoins, reducing the scaffold number by 18.60%, and increasing the scaffold N50 by 0.29%.

**Figure 2.**
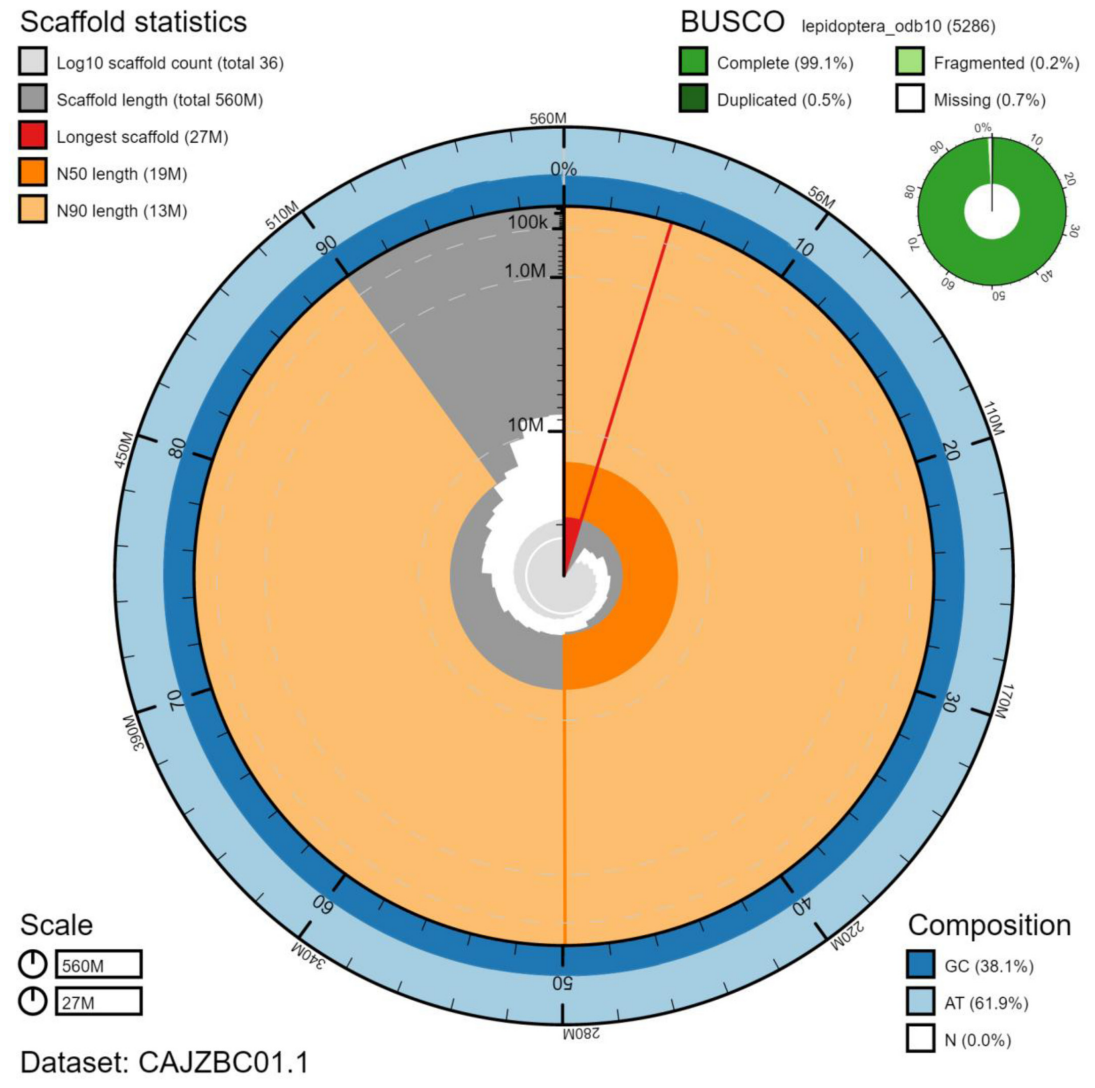
Genome assembly of *Hydraecia micacea*, ilHydMica1.1: metrics. The BlobToolKit Snailplot shows N50 metrics and BUSCO gene completeness. The main plot is divided into 1,000 size-ordered bins around the circumference with each bin representing 0.1% of the 562,441,436 bp assembly. The distribution of chromosome lengths is shown in dark grey with the plot radius scaled to the longest chromosome present in the assembly (26,946,823 bp, shown in red). Orange and pale-orange arcs show the N50 and N90 chromosome lengths (18,862,954 and 12,891,245 bp), respectively. The pale grey spiral shows the cumulative chromosome count on a log scale with white scale lines showing successive orders of magnitude. The blue and pale-blue area around the outside of the plot shows the distribution of GC, AT and N percentages in the same bins as the inner plot. A summary of complete, fragmented, duplicated and missing BUSCO genes in the lepidoptera_odb10 set is shown in the top right. An interactive version of this figure is available at https://blobtoolkit.genomehubs.org/view/ilHydMica1.1/dataset/CAJZBC01.1/snail.

The final assembly has a total length of 562 Mb in 35 sequence scaffolds with a scaffold N50 of 18.9 Mb ( Table 1). The majority of the assembly sequence (99.98%) was assigned to 32 chromosomal-level scaffolds, representing 30 autosomes (numbered by sequence length), and the W and Z sex chromosomes ( Figure 2– Figure 5; Table 2). The assembly has a BUSCO v5.2.2 ( Manni *et al*., 2021) completeness of 99.1% (single 98.6%, duplicated 0.5%) using the lepidoptera_odb10 reference set. While not fully phased, the assembly deposited is of one haplotype. Contigs corresponding to the second haplotype have also been deposited.

**Table 1.** Genome data for *Hydraecia micacea*, ilHydMica1.1.

*Project accession data*	
Assembly identifier	ilHydMica1.1
Species	*Hydraecia micacea*
Specimen	ilHydMica1
NCBI taxonomy ID	NCBI:txid214171
BioProject	PRJEB46321
BioSample ID	SAMEA8603188
Isolate information	Female, thorax (genome assembly), head (Hi-C)
*Raw data accessions*	
PacificBiosciences SEQUEL II	ERR6939243
10X Genomics Illumina	ERR6688535-ERR6688538
Hi-C Illumina	ERR6688534
*Genome assembly*	
Assembly accession	GCA_914767645.1
Accession of alternate haplotype	GCA_914767565.1
Span (Mb)	562
Number of contigs	53
Contig N50 length (Mb)	18.8
Number of scaffolds	35
Scaffold N50 length (Mb)	18.9
Longest scaffold (Mb)	22.0
BUSCO * genome score	C:99.1%[S:98.6%,D:0.5%], F:0.2%,M:0.7%,n:5286

*BUSCO scores based on the lepidoptera_odb10 BUSCO set using v5.2.2. C= complete [S= single copy, D=duplicated], F=fragmented, M=missing, n=number of orthologues in comparison. A full set of BUSCO scores is available at https://blobtoolkit.genomehubs.org/view/ilHydMica1.1/dataset/CAJZBC01.1/busco.

**Figure 3.**
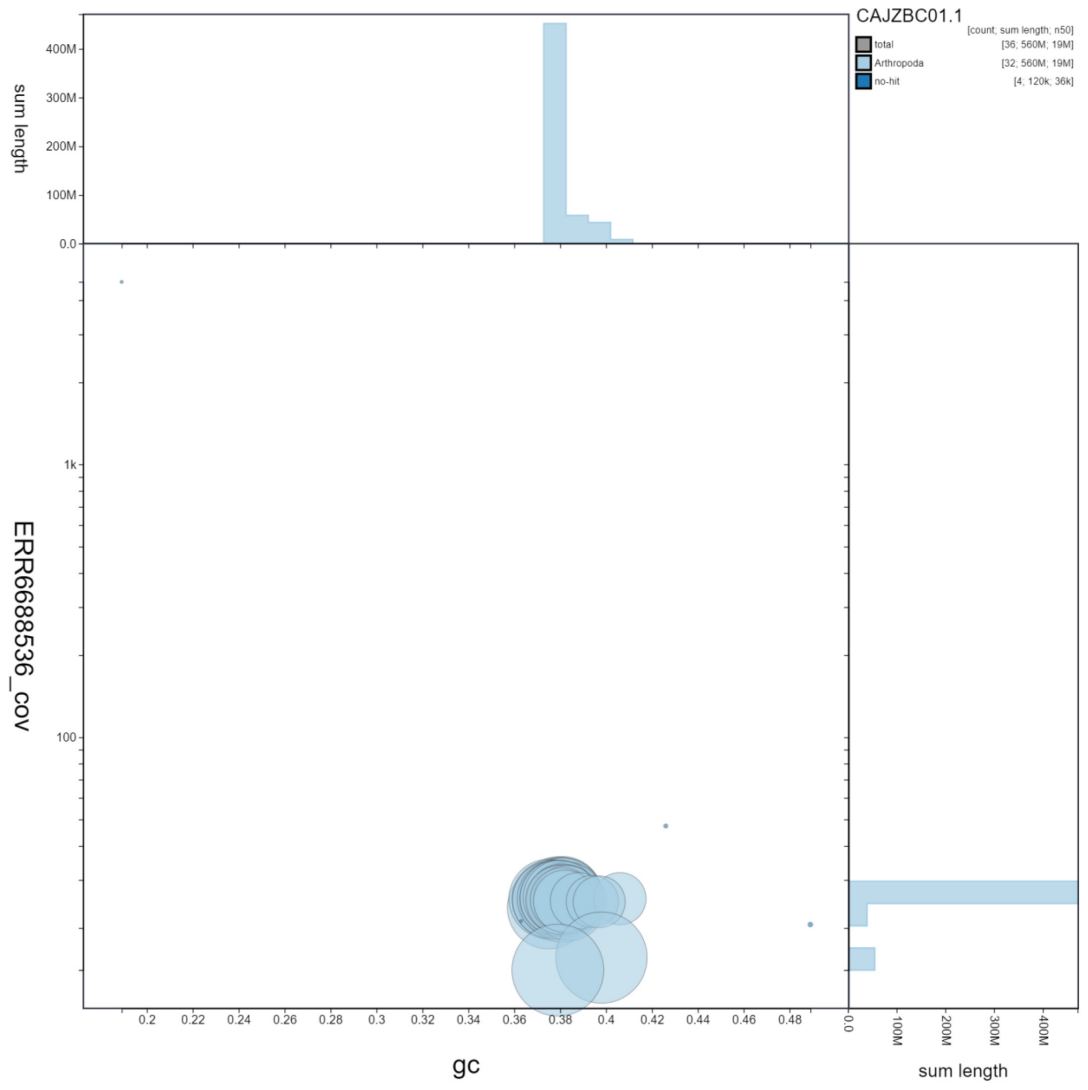
Genome assembly of *Hydraecia micacea*, ilHydMica1.1: GC coverage. BlobToolKit GC-coverage plot. Scaffolds are coloured by phylum. Circles are sized in proportion to scaffold length Histograms show the distribution of scaffold length sum along each axis. An interactive version of this figure is available at https://blobtoolkit.genomehubs.org/view/ilHydMica1.1/dataset/CAJZBC01.1/blob.

**Figure 4.**
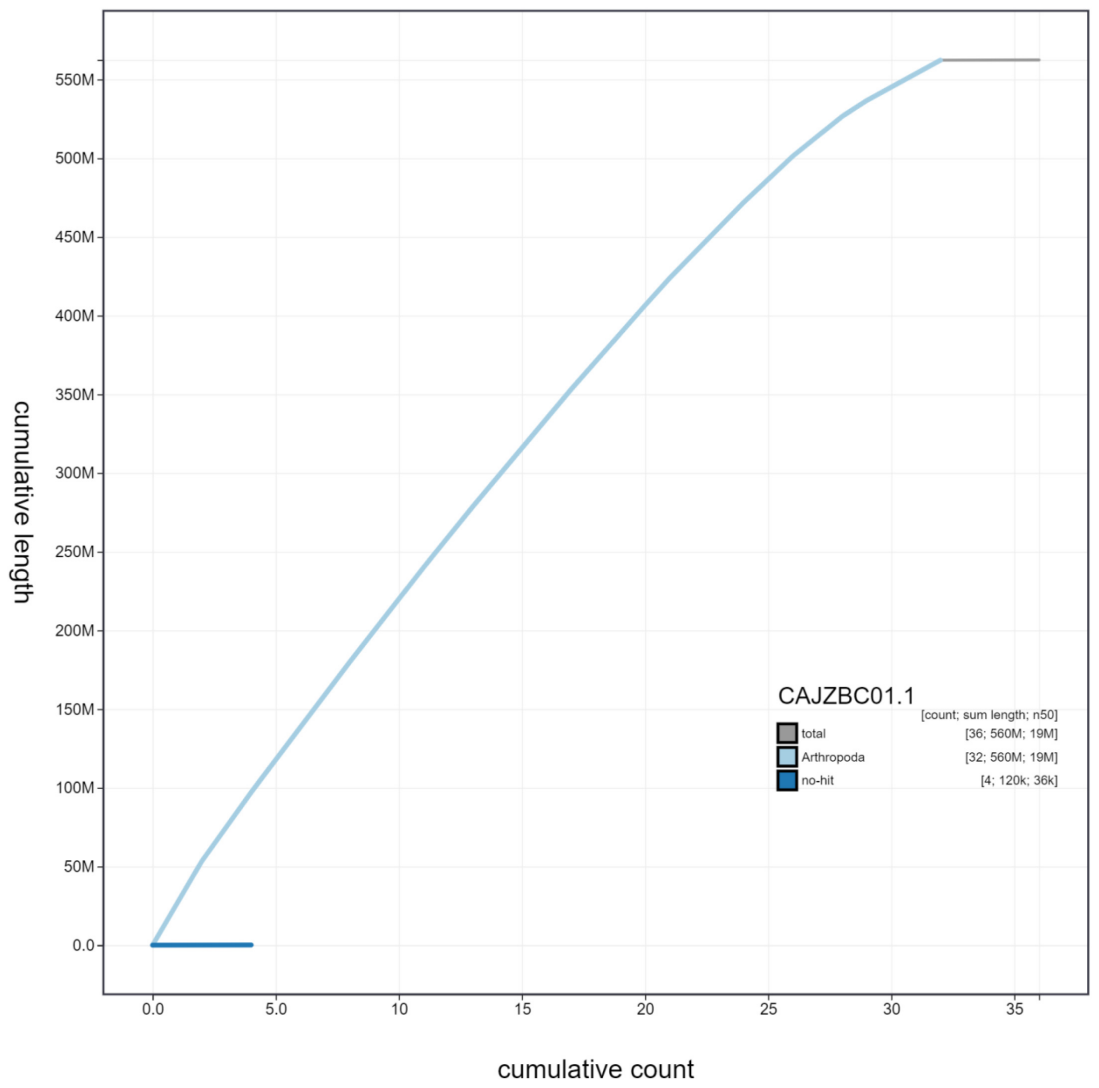
Genome assembly of *Hydraecia micacea*, ilHydMica1.1: cumulative sequence. BlobToolKit cumulative sequence plot. The grey line shows cumulative length for all scaffolds. Coloured lines show cumulative lengths of scaffolds assigned to each phylum using the buscogenes taxrule. An interactive version of this figure is available at https://blobtoolkit.genomehubs.org/view/ilHydMica1.1/dataset/CAJZBC01.1/cumulative.

**Figure 5.**
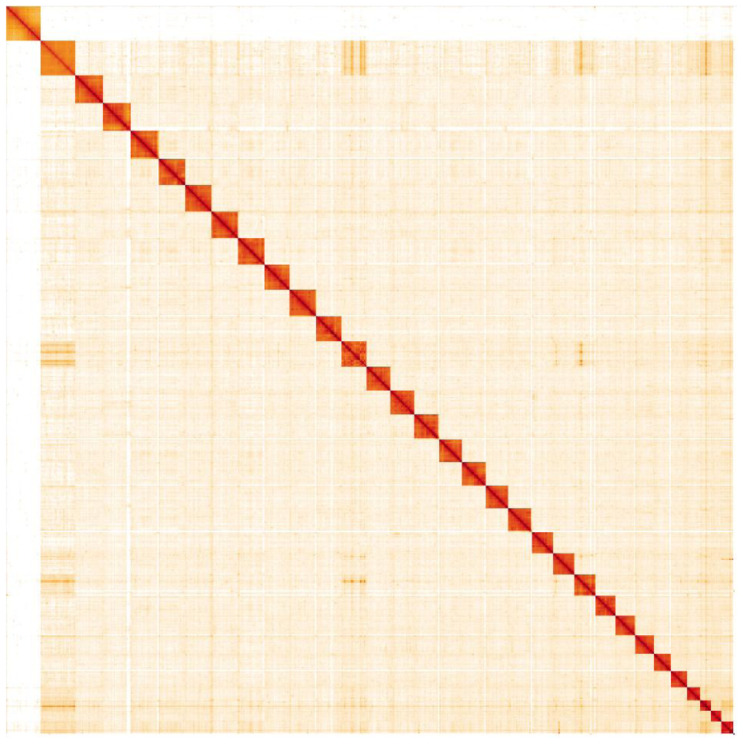
Genome assembly of *Hydraecia micacea*, ilHydMica1.1: Hi-C contact map. Hi-C contact map of the ilHydMica1.1 assembly, visualised in HiGlass. Chromosomes are shown in order of size from left to right and top to bottom. An interactive version of this map is available here.

**Table 2.** Chromosomal pseudomolecules in the genome assembly of *Hydraecia micacea*, ilHydMica1.1.

INSDC accession	Chromosome	Size (Mb)	GC%
OU611776.1	1	21.98	37.9
OU611777.1	2	21.72	37.5
OU611778.1	3	20.85	38.1
OU611779.1	4	20.82	38.1
OU611780.1	5	20.46	38.0
OU611781.1	6	20.40	37.7
OU611782.1	7	20.20	38.1
OU611783.1	8	20.12	37.5
OU611784.1	9	19.96	37.8
OU611785.1	10	19.30	37.6
OU611786.1	11	19.27	38.1
OU611787.1	12	18.86	37.6
OU611788.1	13	18.81	37.9
OU611789.1	14	18.57	37.8
OU611790.1	15	18.34	37.9
OU611791.1	16	18.17	37.9
OU611792.1	17	17.60	38.2
OU611793.1	18	17.58	38.3
OU611794.1	19	17.32	38.0
OU611795.1	20	16.18	38.1
OU611796.1	21	15.95	38.4
OU611797.1	22	15.84	38.2
OU611798.1	23	15.00	38.0
OU611799.1	24	14.57	38.3
OU611800.1	25	12.89	38.1
OU611801.1	26	12.32	38.2
OU611802.1	27	10.04	38.8
OU611803.1	28	8.62	40.5
OU611804.1	29	8.59	39.4
OU611805.1	30	8.48	39.7
OU611775.1	W	26.58	39.8
OU611774.1	Z	26.95	37.9
OU611806.1	MT	0.02	19.2
-	Unplaced	0.10	44.6

## Methods

### Sample acquisition and DNA extraction

A single female *H. micacea* (ilHydMica1) was collected from Wytham Woods, Oxfordshire (Biological vice-county: Berkshire), UK (latitude 51.772, longitude -1.338) by Douglas Boyes, UKCEH, using a light trap in woodland. The sample was identified by the same individual, and preserved on dry ice.

DNA was extracted at the Tree of Life laboratory, Wellcome Sanger Institute. The ilHydMica1 sample was weighed and dissected on dry ice with tissue set aside for Hi-C sequencing. Thorax tissue was cryogenically disrupted to a fine powder using a Covaris cryoPREP Automated Dry Pulveriser, receiving multiple impacts. Fragment size analysis of 0.01–0.5 ng of DNA was then performed using an Agilent FemtoPulse. High molecular weight (HMW) DNA was extracted using the Qiagen MagAttract HMW DNA extraction kit. Low molecular weight DNA was removed from a 200-ng aliquot of extracted DNA using 0.8X AMpure XP purification kit prior to 10X Chromium sequencing; a minimum of 50 ng DNA was submitted for 10X sequencing. HMW DNA was sheared into an average fragment size between 12-20 kb in a Megaruptor 3 system with speed setting 30. Sheared DNA was purified by solid-phase reversible immobilisation using AMPure PB beads with a 1.8X ratio of beads to sample to remove the shorter fragments and concentrate the DNA sample. The concentration of the sheared and purified DNA was assessed using a Nanodrop spectrophotometer and Qubit Fluorometer and Qubit dsDNA High Sensitivity Assay kit. Fragment size distribution was evaluated by running the sample on the FemtoPulse system.

### Sequencing

Pacific Biosciences HiFi circular consensus and 10X Genomics Chromium read cloud sequencing libraries were constructed according to the manufacturers’ instructions. Sequencing was performed by the Scientific Operations core at the Wellcome Sanger Institute on Pacific Biosciences SEQUEL II (HiFi) and Illumina NovaSeq 6000 (10X) instruments. Hi-C data were generated from head tissue of ilHydMica1 using the Arima Hi-C+ kit and sequenced on NovaSeq 6000.

### Genome assembly

Assembly was carried out with Hifiasm ( Cheng *et al*., 2021); haplotypic duplication was identified and removed with purge_dups ( Guan *et al*., 2020). One round of polishing was performed by aligning 10X Genomics read data to the assembly with longranger align, calling variants with freebayes ( Garrison & Marth, 2012). The assembly was then scaffolded with Hi-C data ( Rao *et al*., 2014) using SALSA2 ( Ghurye *et al*., 2019). The assembly was checked for contamination as described previously ( Howe *et al*., 2021). Manual curation ( Howe *et al*., 2021) was performed using HiGlass ( Kerpedjiev *et al*., 2018) and Pretext. The mitochondrial genome was assembled using MitoHiFi ( Uliano-Silva *et al*., 2021), which performs annotation using MitoFinder ( Allio *et al*., 2020). The genome was analysed and BUSCO scores generated within the BlobToolKit environment ( Challis *et al*., 2020). Table 3 contains a list of all software tool versions used, where appropriate.

**Table 3.** Software tools used.

Software tool	Version	Source
Hifiasm	0.15.3	Cheng *et al*., 2021
purge_dups	1.2.3	Guan *et al*., 2020
SALSA	2.2	Ghurye *et al*., 2019
longranger align	2.2.2	https://support.10xgenomics.com/genome-exome/ software/pipelines/latest/advanced/other-pipelines
freebayes	1.3.1-17-gaa2ace8	Garrison & Marth, 2012
MitoHiFi	2.0	Uliano-Silva *et al*., 2021
HiGlass	1.11.6	Kerpedjiev *et al*., 2018
PretextView	0.2.x	https://github.com/wtsi-hpag/PretextView
BlobToolKit	3.0.5	Challis *et al*., 2020

### Ethics/compliance issues

The materials that have contributed to this genome note have been supplied by a Darwin Tree of Life Partner. The submission of materials by a Darwin Tree of Life Partner is subject to the Darwin Tree of Life Project Sampling Code of Practice. By agreeing with and signing up to the Sampling Code of Practice, the Darwin Tree of Life Partner agrees they will meet the legal and ethical requirements and standards set out within this document in respect of all samples acquired for, and supplied to, the Darwin Tree of Life Project. Each transfer of samples is further undertaken according to a Research Collaboration Agreement or Material Transfer Agreement entered into by the Darwin Tree of Life Partner, Genome Research Limited (operating as the Wellcome Sanger Institute), and in some circumstances other Darwin Tree of Life collaborators.

## Data availability

European Nucleotide Archive: Hydraecia micacea (rosy rustic). Accession number PRJEB46321; https://identifiers.org/ena.embl/PRJEB46321.

The genome sequence is released openly for reuse. The *H. micacea* genome sequencing initiative is part of the Darwin Tree of Life (DToL) project. All raw sequence data and the assembly have been deposited in INSDC databases. The genome will be annotated and presented through the Ensembl pipeline at the European Bioinformatics Institute. Raw data and assembly accession identifiers are reported in Table 1.

## Author information

Members of the University of Oxford and Wytham Woods Genome Acquisition Lab are listed here: https://doi.org/10.5281/zenodo.5746938.

Members of the Darwin Tree of Life Barcoding collective are listed here: https://doi.org/10.5281/zenodo.5744972.

Members of the Wellcome Sanger Institute Tree of Life programme are listed here: https://doi.org/10.5281/zenodo.6125027.

Members of Wellcome Sanger Institute Scientific Operations: DNA Pipelines collective are listed here: https://doi.org/10.5281/zenodo.5746904.

Members of the Tree of Life Core Informatics collective are listed here: https://doi.org/10.5281/zenodo.6125046.

Members of the Darwin Tree of Life Consortium are listed here: https://doi.org/10.5281/zenodo.5638618.
