# Supplementary material for: The genome sequence of the rosy rustic, Hydraecia micacea (Esper, 1789)
Source: Wellcome Open Res. 2022 Apr 7;7:131. [Version 1] doi: 10.12688/wellcomeopenres.17832.1 (PMC10632591; doi:10.12688/wellcomeopenres.17832.1)
Supplement: Peer review history

# Reviewer report: Reviewer response for version 1

**DOI:** 10.21956/wellcomeopenres.19744.r68768

**Published:** 2023-11-08

**Authors:** Simon H Martin

**Affiliations:** 1 Institute of Evolutionary Biology, University of Edinburgh, Edinburgh, UK

The report describes a chromosomal assembly for the rosy rustic moth. The approaches used are state of the art, and the resulting assembly is of high quality. I have no scientific concerns with the report.

 I spotted one factual error: Table 1 reports that the longest scaffold is 22 Mb, but it should be 27 Mb (Z chromosome).

 Note that in some places Latin names are not italicised.

Are sufficient details of methods and materials provided to allow replication by others?

Yes

Is the rationale for creating the dataset(s) clearly described?

Yes

Are the datasets clearly presented in a useable and accessible format?

Yes

Are the protocols appropriate and is the work technically sound?

Yes

Reviewer Expertise:

Evolutionary genomics

I confirm that I have read this submission and believe that I have an appropriate level of expertise to confirm that it is of an acceptable scientific standard.

# Reviewer report: Reviewer response for version 1

**DOI:** 10.21956/wellcomeopenres.19744.r68777

**Published:** 2023-10-27

**Authors:** Jason Hill

**Affiliations:** 1 Science for Life Laboratory, Department of Medical Biochemistry and Microbiology, Uppsala University, Uppsala, Sweden

The authors Boyes *et al.* presented a genome assembly of the Rosy Rustic Moth ( *Hydraecia micacea*). The sequencing and assembly process reflect the best practices for Lepidopteran genomes, and the results demonstrate that the assembly is complete and high quality. More detail on the polishing process would have been useful.

Are sufficient details of methods and materials provided to allow replication by others?

Yes

Is the rationale for creating the dataset(s) clearly described?

Yes

Are the datasets clearly presented in a useable and accessible format?

Yes

Are the protocols appropriate and is the work technically sound?

Yes

Reviewer Expertise:

Genome assembly and annotation, comparative genomics, Lepidoptera genomics.

I confirm that I have read this submission and believe that I have an appropriate level of expertise to confirm that it is of an acceptable scientific standard.
